# Tendon vibration attenuates superficial venous vessel response of the resting limb during static arm exercise

**DOI:** 10.1186/1880-6805-31-29

**Published:** 2012-11-07

**Authors:** Anna Ooue, Kohei Sato, Ai Hirasawa, Tomoko Sadamoto

**Affiliations:** 1 Research Institute of Physical Fitness, Japan Women’s College of Physical Education, 8-19-1 Kitakarasuyama, Setagaya-ku, Tokyo 157-8565, Japan; 2 Graduate school of Engineering, Toyo University, 2100 Kujirai, Kawagoe City, Saitama, 350-8585, Japan

**Keywords:** Central command, Ultrasound technique, Venoconstriction, Venous return

## Abstract

**Background:**

The superficial vein of the resting limb constricts sympathetically during exercise. Central command is the one of the neural mechanisms that controls the cardiovascular response to exercise. However, it is not clear whether central command contributes to venous vessel response during exercise. Tendon vibration during static elbow flexion causes primary muscle spindle afferents, such that a lower central command is required to achieve a given force without altering muscle force. The purpose of this study was therefore to investigate whether a reduction in central command during static exercise with tendon vibration influences the superficial venous vessel response in the resting limb.

**Methods:**

Eleven subjects performed static elbow flexion at 35% of maximal voluntary contraction with (EX + VIB) and without (EX) vibration of the biceps brachii tendon. The heart rate, mean arterial pressure, and rating of perceived exertion (RPE) in overall and exercising muscle were measured. The cross-sectional area (CSA_vein_) and blood velocity of the basilic vein in the resting upper arm were assessed by ultrasound, and blood flow (BF_vein_) was calculated using both variables.

**Results:**

Muscle tension during exercise was similar between EX and EX + VIB. However, RPEs at EX + VIB were lower than those at EX (*P* <0.05). Increases in heart rate and mean arterial pressure during exercise at EX + VIB were also lower than those at EX (*P* <0.05). CSA_vein_ in the resting limb at EX decreased during exercise from baseline (*P* <0.05), but CSA_vein_ at EX + VIB did not change during exercise. CSA_vein_ during exercise at EX was smaller than that at EX + VIB (*P* <0.05). However, BF_vein_ did not change during the protocol under either condition. The decreases in circulatory response and RPEs during EX + VIB, despite identical muscle tension, showed that activation of central command was less during EX + VIB than during EX. Abolishment of the decrease in CSA_vein_ during exercise at EX + VIB may thus have been caused by a lower level of central command at EX + VIB rather than EX.

**Conclusion:**

Diminished central command induced by tendon vibration may attenuate the superficial venous vessel response of the resting limb during sustained static arm exercise.

## Background

Venomotor response is considered to play an important role in the transfer of blood from veins to the heart. Many studies have suggested that sympathetic activation has an impact on the venomotor responses in the resting limb during static exercise [1-3] and dynamic exercise [1,4-6], since especially superficial venous vessels have rich innervation of sympathetic nerves [7,8] and an operation of sympathectomy [1] and a dosage of α-blocking agent clearly abolishes the venoconstriction [2] observed during exercise.

Sustained static exercise produces significant activation of the sympathetic nervous system, and the sympathetic activation during exercise is governed by both central (that is, central command) and peripheral (that is, muscle metaboreflex and mechanoreflex) mechanisms [9-16]. Metabolically sensitive afferents within exercising skeletal muscle detect the buildup of metabolites and act through cardiovascular centers to produce a muscle metaboreflex [11,13]. A component of this muscle reflex may arise from muscle mechanoreflex afferents [10]. Duprez and colleagues reported that post-exercise muscle ischemia produces a significant decrease in venous volume in the contralateral limb, and consequently suggested the importance of muscle metaboreflex on venomotor tone in the non-exercising limb during exercise [17].

In addition to muscle metaboreflex, an essential central command role has emerged from a preliminary study [2]. The concept of central command has been conventionally defined as feed-forward control. When a motor command is sent to a muscle, a parallel or collateral command is sent to cardiovascular centers in the brainstem, and this acts to activate sympathetic nerve activity. Indeed, Lorentsen found an anticipatory increase in the venous pressure in the contralateral limb before the onset of exercise [2], indicating that feed-forward control of the central command plays an important role in venous tone. However, the study did not examine the influence of central command during actual exercise [2]. Recent studies have also reported that central command also functions as feedback control, in which somatosensory signals arising from the working muscles continuously provide a feedback signal and probably modulate cardiovascular responses via alterations of perception of effort or effort sense [18-20]. If central command has the function of feedback control, the influence of central command will appear not only before exercise but also during sustained and later periods of exercise. However, this question has not yet been challenged. Verifying the influence of central command on the venous tone during actual and sustained static exercise is therefore necessary. On the other hand, using the non-invasive ultrasound Doppler method [21-24], assessment of a single vein response during exercise can extend prior knowledge about the influence of central command on the venous system during exercise.

Based on these considerations, we investigated whether central command affects venomotor tone in the contralateral limb during sustained static exercise. In the present study, using vibration of the biceps brachii tendon reported previously [14,25], we evaluated whether less activation of central command during sustained static elbow flexion accompanies lower responses of the cross-sectional area of the superficial vein in the resting upper arm (CSA_vein_). Tendon vibration during active muscle contraction excites the primary afferents of muscle spindles of the contracting muscle, thereby inducing reflex tension via the monosynaptic tendon reflex, which in turn aids voluntary tension development and consequently reduces the amount of central command required to generate a given force [14,25,26]. In addition, tendon vibration was also useful for investigating the influence of central command without inducing discomfort or nociceptor afferent input [14].

## Methods

### Subjects

Eleven healthy subjects (three males and eight females) volunteered to participate in the study. Their mean ± standard deviation age, height, and weight were 21.3 ± 0.9 years, 165.0 ± 6.6 cm, and 55.2 ± 5.7 kg, respectively. All subjects were nonsmokers. The participants were asked not to drink beverages containing caffeine or alcohol for 24 hours and not to eat for at least 2 hours before the start of the experiment. The purpose, procedures, and risks of the study were explained to the subjects, and their informed consent was obtained. The study was approved by the Human Ethics Committee of the Japan Women’s College of Physical Education and was conducted in accordance with the Declaration of Helsinki.

### Muscle tendon vibration and maximal voluntary contraction

Before the main protocol, the subjects were examined to establish the force produced by tendon vibration at rest. A custom vibrator (DPS-380; Dia Medical, Tokyo, Japan) was used to induce left biceps brachii muscle contraction by reflex stimulation of the biceps brachii distal tendon on the cubital fossa [26]. The oscillating frequency of the vibrator was 100 Hz and its amplitude was 0.8 mm. On the same day, the subjects performed two maximal voluntary static elbow flexions of the left arm using a computer-based multifunctional dynamometer (VINE, Tokyo, Japan) to determine their maximal voluntary contraction (MVC) strength, defined as the highest value obtained in the two trials.

### Experimental protocol

In a room maintained at 25.1 ± 0.2°C, each subject stayed in a semi-reclined position in a chair in which body position could be maintained, while the left elbow was kept at a 90° angle on a padded armrest with the wrist attached to an arm lever by a Velcro strap. The subjects rested for at least 20 minutes before data collection began. After baseline data were collected for 5 minutes, subjects performed: static elbow flexion at 35% MVC without vibration of the biceps tendon for 2 minutes (EX); and static elbow flexion at 35% MVC with vibration of the biceps tendon for 2 minutes (EX + VIB). Each exercise period was followed by a recovery period of 1 minute. Static elbow flexion was produced using the same dynamometer that was used to measure the MVC (VINE), with visual feedback of the achieved force provided via an oscilloscope display. For EX + VIB, tendon vibration was initiated 1 minute before starting exercise and continued during the exercise. Immediately after exercise, subjects read instructions for the 6 to 20 rating of perceived exertion (Overall RPE) category scale developed by Borg [27] and instructions for rating muscle fatigue sensation (Arm RPE) on a scale of 1 to 10 [28]. In all trials, subjects regulated their respiratory frequency at 10 or 15 breaths/minute using a metronome, because exercise movement and respiratory cycle influence sympathetic nervous system activity. EX and EX + VIB were performed randomly, and the rest period between the two conditions was at least 20 minutes.

### Measurements

Beat-to-beat changes in arterial pressure were assessed by finger photoplethysmography (Finometer; Finapres Medical Systems BV, Arnhem, the Netherlands). The monitoring cuff was placed around the middle finger. The heart rate (HR) and mean arterial pressure (MAP) were determined from the blood pressure waveform using the Modelflow software program, taking into account sex, age, height, and weight (BeatScope 1.1; Finapres Medical Systems BV).

Muscle oxygenation (oxyhemoglobin (oxy-Hb) and deoxyhemoglobin (deoxy-Hb) concentration) in the left exercising upper arm and right resting forearm was monitored using a near-infrared spectroscopy system (NIRO-200; Hamamatsu Photonics, Hamamatsu, Japan) at dual wavelengths (760 nm and 850 nm). The near-infrared spectroscopy probe consisted of an optically dense holder containing an emission and detection probe and was secured to the skin with tape to minimize extraneous light.

To measure blood velocity (V_vein_) and cross-sectional area (CSA), non-invasive ultrasound imaging of the basilic vein (superficial vein) of the resting upper arm was performed 5 to 6 cm proximal to the cubitus using an 8.7-MHz linear array transducer (Vivid e; GE Healthcare Japan, Tokyo, Japan). A large quantity of ultrasound transmission gel was used to prevent direct contact with the skin and to avoid compression of the vein. V_vein_ and CSA were simultaneously measured on a transverse scan of the vein with the transducer tilted at 60°. Positioning of the transducer was determined at the beginning of each experiment, and it remained unchanged to limit potential errors in Doppler angle. V_vein_ was the result of the mean velocity of spectral Doppler recording every 12 seconds. CSA was calculated by manually tracing the edge of the offline transverse venous image at an arbitrary three points every 12 seconds, and then the three CSA values were averaged. Because CSA was obtained from the image measured at 60°, an accurate CSA (CSA_vein_) was determined as follows:

(1) $${\text{CSA}}_{\text{vein}}\left({\text{cm}}^{2}\right)=\text{CSA}\times \text{sin}\;60{}^{\circ}$$

BF_vein_ in the basilic vein was calculated according to the following formula:

(2) $${\text{BF}}_{\text{vein}}\left(\text{ml}/\text{min}\right)={\text{V}}_{\text{vein}}\times {\text{CSA}}_{\text{vein}}$$

### Data analysis and statistical analysis

The HR, MAP, muscle oxygenation, CSA_vein_, V_vein_, and BF_vein_ were averaged for 61 to 240 seconds before commencing exercise to establish a baseline value. The relative change in these variables from baseline during exercise and the recovery period was calculated. Data are expressed as mean ± standard error values.

To compare the time-course changes, two-way analysis of variance with repeated measures was applied to the circulatory responses, CSA_vein_, V_vein_, and BF_vein_ under each condition (EX and EX + VIB), using time and condition as fixed factors. If a main effect of condition and/or interaction was detected, *post hoc* analysis with a paired *t* test was performed; and if a main effect of time was detected, *post hoc* analysis with a Bonferroni test was performed. To compare the baseline data of the circulatory response, CSA_vein_, V_vein_, and BF_vein_ between EX and EX + VIB, a paired *t* test was performed. In addition, differences in Overall RPE and Arm RPE between conditions were evaluated by paired *t* test. *P* <0.05 was considered significant.

## Results

Vibration of the biceps tendon for 2 minutes elicited a reflex force equivalent to 5.3 ± 2.3% of MVC. However, the HR (from 63 ± 3 beats/minute to 64 ± 2 beats/minute), MAP (from 78 ± 3 mmHg to 78 ± 3 mmHg), CSA_vein_ (from 0.20 ± 0.03 cm^2^ to 0.20 ± 0.03 cm^2^), V_vein_ (from 4.1 ± 0.5 cm/second to 3.9 ± 0.5 cm/second), and BF_vein_ of the basilic vein (from 51.6 ± 10.3 ml/minute to 52.7 ± 11.9 ml/minute) did not change.

There were no significant differences in the baseline data of circulatory responses, CSA_vein_, V_vein_, and BF_vein_ between EX and EX + VIB (Table 1).

**Table 1:** Baseline data under each condition

	**EX**	**EX + VIB**
Heart rate (beats/minute)	62 ± 3	64 ± 3
Mean arterial pressure (mmHg)	84 ± 3	79 ± 3
Venous cross-sectional area (cm^2^)	0.19 ± 0.03	0.20 ± 0.03
Venous blood velocity (cm/second)	3.3 ± 0.3	3.6 ± 0.3
Venous blood flow (ml/minute)	39.2 ± 7.3	47.4 ± 8.4

Values presented as mean ± standard error. Static elbow flexion at 35% of maximal voluntary contraction with (EX + VIB) and without (EX) vibration of the biceps brachii tendon.

**Figure 1:**
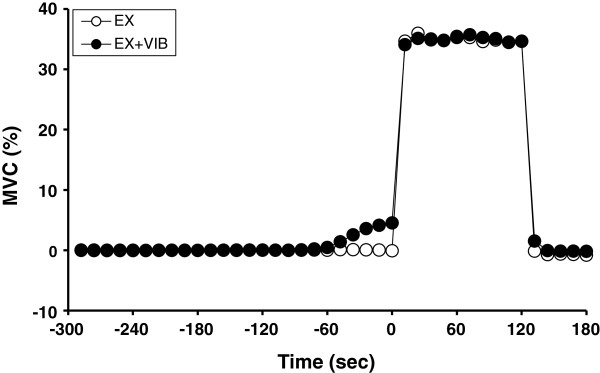
**Time courses of muscle tension under each condition.**Static elbow flexion at 35% of maximal voluntary contraction with (EX + VIB) and without (EX) vibration of the biceps brachii tendon. Data expressed as mean ± standard error.

Muscle tension during EX was similar to that during EX + VIB (Figure 1). However, both Overall RPE and Arm RPE after EX + VIB were significantly lower than after EX (Overall RPE: 11.5 ± 0.2 vs. 12.6 ± 0.3, *P* <0.05; Arm RPE: 3.2 ± 0.3 vs. 4.9 ± 0.4, *P* <0.05).

**Figure 2:**
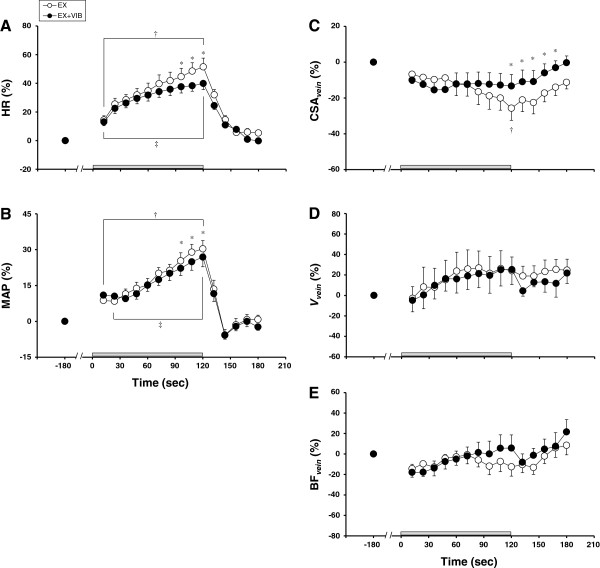
**Relative changes in circulatory responses and blood flow responses in the resting upper arm.** Relative changes in (**A**) heart rate (HR), (**B**) mean arterial pressure (MAP), (**C**) cross-sectional area (CSA_vein_), (**D**) blood velocity (V_vein_), and (**E**) venous blood flow (BF_vein_) of the basilic vein in the resting upper arm during static elbow flexion at 35% of maximal voluntary contraction with (EX + VIB) and without (EX) vibration of the biceps brachii tendon. Data expressed as mean ± standard error. *P <0.05, difference between EX and EX + VIB; †*P* <0.05, difference from baseline level during EX; ‡*P* <0.05, difference from baseline level during EX + VIB.

Figure 2 shows the time courses of HR, MAP, and CSA_vein_, V_vein_, and BF_vein_ of the resting upper arm during EX and EX + VIB. The increase in HR during exercise from 96 to 120 seconds at EX + VIB was less than that at EX (values at 120 seconds of exercise: 39.1 ± 4.0% vs. 50.0 ± 5.9%, *P* <0.05) (Figure 2A). Likewise, the increase in MAP during exercise from 96 to 120 seconds at EX + VIB was lower than that at EX (values at 120 seconds of exercise: 26.0 ± 3.7% vs. 29.6 ± 3.2%, *P* <0.05) (Figure 2B). CSA_vein_ during exercise at EX decreased from baseline (values at 120 seconds of exercise: –22.9 ± 6.7%, *P* <0.05), but CSA_vein_ during EX + VIB did not change from baseline throughout the protocol. In addition, CSA_vein_ at 120 seconds of exercise and during recovery at EX was lower than at EX + VIB (*P* <0.05) (Figure 2C). V_vein_ and BF_vein_ did not change from baseline, and this response was similar under both conditions (Figure 2D,E).

**Figure 3:**
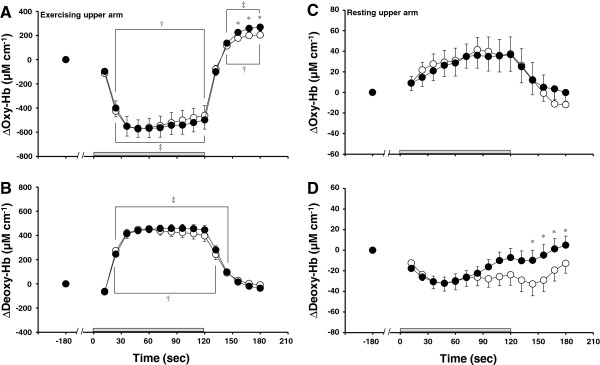
**Relative changes in muscle oxygenation of the exercising upper arm and resting forearm.** Relative changes in the oxyhemoglobin (Δoxy-Hb) and deoxyhemoglobin (Δdeoxy-Hb) concentrations of the (**A, B**) exercising upper arm and (**C, D**) resting forearm during static elbow flexion at 35% of maximal voluntary contraction with (EX + VIB) and without (EX) vibration of the biceps brachii tendon. Data expressed as mean ± standard error. *P <0.05, difference between EX and EX + VIB; †*P* <0.05, difference from baseline level during EX; ‡*P* <0.05, difference from baseline level during EX + VIB.

Figure 3 shows the time courses of muscle oxygenation of the exercising upper arm and resting forearm during EX and EX + VIB. In the exercising upper arm, Δoxy-Hb decreased from baseline during exercise and increased from baseline during recovery in both EX and EX + VIB (*P* <0.05). Δoxy-Hb of the exercising upper arm during recovery at EX was lower than at EX + VIB (*P* <0.05; Figure 3A). In the exercising upper arm, Δdeoxy-Hb increased from baseline during exercise (*P* <0.05) and returned to baseline during recovery in both EX and EX + VIB (Figure 3B). In the resting forearm, Δoxy-Hb and Δdeoxy-Hb did not change from baseline at both EX and EX + VIB (Figure 3C,D). However, Δdeoxy-Hb of the resting forearm during recovery at EX was lower than at EX + VIB (*P* <0.05).

## Discussion

The primary findings in this study were that CSA_vein_ decreased from baseline during static elbow flexion alone, although CSA_vein_ during static elbow flexion with tendon vibration did not change, and that BF_vein_ did not change significantly during static exercise with or without tendon vibration. These results suggest that a reduction in central command during static exercise with tendon vibration may attenuate the superficial venous vessel response of the resting limb during sustained static arm exercise.

Superficial venous vessel response may be controlled by both the sympathetic nervous system [1,4,6,29] and changes in venous pressure related to alterations in blood flow and blood volume [30,31]. In our study, BF_vein_ did not change throughout the protocol during EX and EX + VIB (Figure 2E). In addition, Δdeoxy-Hb in the resting forearm did not change from baseline with static elbow flexion during both EX and EX + VIB (Figure 3D). Because the change in oxy-Hb and deoxy-Hb is used to evaluate blood volume in arterial and venous vascular beds, respectively [32,33], it is speculated that the venous blood volume in the resting forearm was unchanged during static elbow flexion. In our study, therefore, the decrease in CSA_vein_ with exercise during EX may have been caused by sympathetic nervous system control. On the other hand, the difference in CSA_vein_ between EX and EX + VIB during the recovery period might have been influenced by the change in venous blood volume but not by the sympathetic nervous system, because Δdeoxy-Hb of the resting forearm was also different between the two conditions (Figure 3D).

The concept of central command has been classically defined as a feed-forward control. Feed-forward characterization may be largely based on the immediate cardiovascular response to onset (or even anticipation) of exercise. In addition to feed-forward control, there is evidence that the effects of central command on cardiovascular responses are closely related to the intensity or perceived effort of the exercise [34,35]. Central command is there also proposed to be capable of functioning as feedback control, in which somatosensory signals arising from the working muscles may provide a feedback signal capable of influencing central command via alterations of perception of effort or effort sense [19,20]. The experimental model in our study might reflect central command that is defined as feedback control rather than feed-forward control, because the changes in HR and MAP, which are indexes of the cardiovascular response, were significantly lower during 96 to 120 seconds of exercise in EX + VIB than in EX (Figure 2A,B). These results are in agreement with those of previous studies [14,25,26]. In addition, the magnitude of the central command response has been assessed using an individual’s perception of effort sense during exercise, independent of force production [15,34]. Although the relationship between central command and RPE has not been clearly defined, the RPE scale [27] has been widely used to assess the level of central command. In the present study, RPE immediately after exercise was lower in EX + VIB than in EX, indicating that central command, which is defined as feedback control, might be lower in EX + VIB than in EX. Thus, related to the central command response that is defined as feedback control, CSA_vein_ was also smaller in EX than in EX + VIB during the latter half of the exercise. In addition, activation of the central command at the onset of static elbow flexion exercise in the present study, which indicated the feed-forward control, may have been too small to cause venoconstriction. If activation of central command at the onset of static elbow flexion exercise was enough to cause venoconstriction, the decrease in CSA_vein_ had to be obtained at the onset of exercise in both EX and EX + VIB.

Vibration is a powerful stimulus for primary muscle spindle afferents when applied to the biceps tendon during static exercise. When the biceps brachii was contracting, activation of its muscle spindle primary afferents provided reflex activation, which in turn aided voluntary tension development compared with contraction only of the biceps brachii. The afferent input of decreased voluntary tension during exercise with tendon vibration might thus cause interactions between perception of effort and central command, such that the activation of central command might alter [20].

The increase in sympathetic nervous system activity during exercise is caused not only by central command but also by the reflex neural mechanism that is activated by exercise (muscle mechanoreflex and muscle metaboreflex) [9-11,13,16]. Muscle-exerted tension during static elbow flexion did not differ between EX and EX + VIB (Figure 1), showing that the degree of activation of muscle mechanoreflex may be similar under both conditions. In addition, Δdeoxy-Hb concentration of the exercising upper arm was similar between EX and EX + VIB (Figure 3B). Because deoxy-Hb of exercising muscle is the index for oxygen consumption [36,37], the level of an exercise-induced metabolite accumulation during EX was expected to be equal to that found during EX + VIB, suggesting that the degree of activation of the muscle metaboreflex might not differ between EX and EX + VIB. In the present study, therefore, it is likely that the difference in CSA_vein_ during static exercise between EX and EX + VIB might not be due to the differences in activation of the reflex neural mechanism under different conditions.

Although the specific regions of the brain involved in exercise-related responses remain speculative, the following theory can be considered. Animal studies suggest that subthalamic regions are capable of generating both motor and cardiovascular responses [38]. In human studies, possible sites and neurocircuitry involving the insular cortex, sensorimotor cortex, anterior cingulate gyrus, medial prefrontal region and thalamic regions [18,39-43], and the periaqueductal gray [44,45], have been suggested. In addition, a recent hypothesis concerning the neural circuit responsible for generating central command is as follows: cerebral cortical output is not an essential component for the generation of central command but does seem to require a process that triggers activity in neural circuit(s) in the caudal brain to generate central command, and the region from the caudal diencephalon to the rostral mesencephalon plays an important role in the generation of central command [46], because in the decerebrate animal study the renal sympathetic nerve activity and HR abruptly increased in association with the start of locomotion [47], and spontaneous motor activity and the associated cardiovascular response were lost after decerebration at the midcollicular level [48].

Stewart and colleagues reported that venoconstriction during static exercise, which occurs not only in the splanchnic area but also in the resting extremities, may contribute to an increase in venous return to the heart to increase cardiac output [49]. Taking into account previous studies, including our own, venoconstriction via central command might play a significant role in hemodynamics during exercise. However, because the relationship between venous return and venoconstriction is not obvious, further investigation is required.

### Limitations

Several limitations should be considered when interpreting our results. First, due to the large compliance of veins, volume (that is, CSA_vein_) is dependent on the venous pressure level – but we did not measure venous pressure. As mentioned above, however, BF_vein_ and Δdeoxy-Hb (an index of venous blood volume) of the resting forearm did not change from baseline during both EX and EX + VIB (Figures 2E and 3D). We therefore believe that the effect of venous pressure-dependent control was scarcely observed during exercise in this study. Second, we did not account for the menstrual cycle in female subjects. However, because EX and EX + VIB were carried out in same day, this effect may be negligible in our study.

## Conclusions

Static elbow flexion with vibration of the biceps brachii tendon, which caused a decrease in central command during exercise, inhibited the increase in circulatory response and the decrease in CSA_vein_ in the resting upper arm when compared with static exercise alone, although BF_vein_ was similar during exercise both with and without tendon vibration. These findings suggest that central command may contribute to the superficial venous vessel response of the resting limb during sustained static elbow flexion.

## Abbreviations

BF_vein_: Blood flow of the basilic vein; CSA: Cross-sectional area of basilic vein before correction; CSA_vein_: Accurate cross-sectional area of the basilic vein after correction; Δ: Change; EX: Elbow flexion without vibration; EX + VIB: Elbow flexion with vibration; oxy-Hb: Oxyhemoglobin; deoxy-Hb: Deoxyhemoglobin; HR: Heart rate; MAP: Mean arterial pressure; MVC: Maximal voluntary contraction; RPE: Rating of perceived exertion; V_vein_: Blood velocity of the basilic vein.

## Competing interests

The authors declare that they have no competing interests.

## Authors’ contributions

AO designed and coordinated the study, carried out the experiment, and drafted the manuscript. KS participated in the design of the study and helped draft the manuscript. AH helped carry out the experiment. TS participated in the design of the study and helped draft the manuscript. All authors read and approved the final manuscript.
